# A Persian Workman

**Published:** 1885-12

**Authors:** 


### A PERSIAN WORKMAN.

From the illustrated paper by S. G. W. Benjamin, our late minister to Persia, in the city of Teheran, we quote the following: "That implements they used in ancient times we know not; but to-day the Persian artisan has neither rule, compass, nor spirit-level. He is commonly ignorant of the fact that the diameter is the third of the circumference; his gimlets and augurs are prods turned by a bow-string; he has no hatchet, but only an adze, and no carpenter's bench. If he desires to plane a board, he puts it on the ground; and if he would saw a block of wood, he squats on the ground himself and holds it between his toes, drawing the saw towards himself. Wood is scarce, and with such tools hard to work. If pillars are to be constructed, the trunks of poplars are raised and simply stripped of their branches and bark. They may be crooked, but that matters not; the master workman tells his subordinate to shape the the timber into an elegant pillar with *gatch*. Depending only on his eye and the skill of hand, this simple artisan applies the plaster around the trunk in the form of a fluied pillar and crowns it with a graceful capital and cornice, showing a lively inventive fancy. If judged by the strict application of rule and compass, these decorations may sometimes deviate slightly from a straight line, but of the artistic beauty of the conception there can be no question. Walls and ceilings are tastefully decorated in like manner.

* * * "Lightness combined with strength is often gained in Persia by building a wall of square sundried bricks, ingeniously arranged in hollow cubes as in a block-house. They are cemented together by a layer of *cargel*, or mortar mixed with straw, over which, in turn, follows a coat of white plaster. Where great strength is required the angles are fortified by a layer of burnt bricks. Such a wall will stand for ages. It is interesting to watch the builders at work. They wear long tunics, which are tucked into their girdles when working, displaying a length and muscular development of limb I have never seen equaled elsewhere. The one above sings out in musical tone, 'Brother, in the name of God, toss me a brick.' The one below, as he throws the brick, sings in reply, 'O my brother (or, O son of my uncle), in the name of God, behold a brick!'"

---

A RECIPE for the mucilage used on postage stamps calls for:

| | | |
|---|---|---|
| Gum dextrine | 2 | parts. |
| Acetic acid | 1 | " |
| Water | 5 | " |

dissolved in a water bath and 1 part of alcohol added.—*Scien. American.*

